# Regulation of microsomal prostaglandin E_2 _synthase-1 and 5-lipoxygenase-activating protein/5-lipoxygenase by 4-hydroxynonenal in human osteoarthritic chondrocytes

**DOI:** 10.1186/ar2926

**Published:** 2010-02-09

**Authors:** Shu-Huang Chen, Hassan Fahmi, Qin Shi, Mohamed Benderdour

**Affiliations:** 1 Orthopaedic Research Laboratory, Hôpital du Sacré-Caeur de Montréal, Department of Surgery, University of Montreal, 5400 Gouin Blvd West, Montreal, QC H4J 1C5, Canada

## Abstract

**Introduction:**

This study aimed to investigate whether hydroxynonenal (HNE) depletion is responsible for the switch from cyclooxygenase-2 (COX-2) and microsomal prostaglandin E_2 _synthase-1 (mPGES-1) to 5-lipoxygenase-activating protein (FLAP) and 5-lipoxygenase (5-LOX).

**Methods:**

For COX-2 and mPGES-1 studies, human osteoarthritic chondrocytes were stimulated at different incubation times (up to 24 hours) with a single or repetitive addition of 10 μM HNE to the cultures at 2-hour intervals, up to 14 hours. For 5-LOX and FLAP studies, cells were treated with a single addition of 10 μM HNE for 24 hours, 48 hours, and 72 hours in the presence or absence of naproxen (a nonspecific COX-2 inhibitor) or antibody anti-transforming growth factor-beta 1 (TGF-β1). The protein levels of COX-2, mPGES-1 and early growth response factor-1 (Egr-1) transcription factor were evaluated by western blot, and those of prostaglandin E_2 _(PGE_2_), leukotriene B_4 _(LTB_4_) and TGF-β1 were determined with commercial kits. The levels of mPGES-1, FLAP and 5-LOX mRNA were measured by real-time RT-PCR. Transient transfection was performed to determine promoter activities of mPGES-1 and 5-LOX.

**Results:**

Single addition of 10 μM HNE to cultured chondrocytes induced PGE_2 _release as well as COX-2 and mPGES-1 expression at the protein and mRNA levels, with a plateau reached respectively at 8 and 16 hours of incubation, followed by a subsequent decline. However, repeated treatments with HNE prevented the decline of COX-2 and mPGES-1 expression that occurred with a single aldehyde addition. HNE induced mPGES-1 promoter activity, possibly through transcription factor Egr-1 activation. After 48 hours, when COX-2 expression decreased, the LTB_4 _level rose through 5-LOX and FLAP upregulation. The addition of naproxen to cultured chondrocytes revealed that FLAP and 5-LOX regulation by HNE required PGE_2 _production. Furthermore, our data showed that HNE significantly induced TGF-β1 production. The addition of anti-TGF-β1 antibody reduced HNE-induced 5-LOX and FLAP expression by 40%, indicating the partial involvement of a TGF-β1-dependent mechanism.

**Conclusions:**

Our data demonstrate that the shunt to the FLAP and 5-LOX pathway in HNE-induced human osteoarthritic chondrocytes is attributed to COX-2 and mPGES-1 inhibition, probably due to HNE depletion. PGE_2 _and TGF-β1 are suggested to be involved in this regulation.

## Introduction

Osteoarthritis (OA) is a degenerative joint disease with abnormal alterations in the structure, composition and function of articular tissues. Morphological changes in OA include cartilage destruction, osteophyte formation and synovial inflammation [1]. It is believed that overproduced proinflammatory cytokines, such as IL-1β and TNFα, are involved in the pathogenesis of OA by metalloproteinase (MMP) upregulation and collagen downregulation. Moreover, IL-1β and TNFα stimulate the production of prostaglandins, such as prostaglandin E_2 _(PGE_2_), whose roles in the inflammatory process and proteoglycan degradation in human OA cartilage have been reported [2].

PGE_2_, a major prostaglandin produced via arachidonic acid (AA) metabolism, is involved in many physiological events, such as cell growth, immune regulation, inflammation and arthritis [3]. Cyclooxygenase (COX) and prostaglandin E_2 _synthase (PGES) are key enzymes for PGE_2 _biosynthesis under inflammatory conditions. COX-1 and COX-2 convert AA into prostaglandin H_2_, which is subsequently converted to PGE_2 _by PGES. Among all forms of PGES, microsomal PGES-1 (mPGES-1) has been studied the most because of its inducible characteristic and collaboration with inducible COX-2, leading to PGE_2 _production. It has been shown that mPGES-1 expression is upregulated in animal models of rheumatoid arthritis and in patients suffering from OA [4,5] and is downregulated by anti-inflammatory drugs [6,7]. mPGES-1 expression is regulated by several transcription factors, such as early growth response-1 (Egr-1) and NF-κB, in different cell types [8,9].

Leukotrienes, other end-products of AA metabolism, are potent mediators of inflammation that increase the activation, migration and adhesion of immune cells [10]. In particular, leukotriene B_4 _(LTB_4_) promotes the production and release of proinflammatory cytokines from synovial membranes, drawing more attention to its role in OA. Clinical investigations have revealed that long-term COX-2 inhibition causes a switch to the 5-lipoxygenase (5-LOX) pathway, leading to LTB_4 _production [11]. It is now clear that prostaglandins and leukotrienes have additional outcomes; blocking the production of these mediators might therefore have synergistic effects and achieve optimal anti-inflammatory activity [3]. Functional 5-LOX requires binding to 5-lipoxygenase-activating protein (FLAP), which helps 5-LOX binding to AA at the nuclear membrane and enhances the efficiency of leukotriene synthesis [12]. 5-LOX gene expression at the transcriptional level is regulated by several transcription factors, such as smad3/4 and vitamin D receptors [13,14].

Reactive oxygen species produced inside the joints also are associated with the pathogenesis of OA [15]. They induce lipid peroxidation of membrane polyunsaturated fatty acids, eliciting the production of aldehydes from AA [16,17]. 4-Hydroxynonenal (HNE) has been found to be the predominantly produced and most reactive aldehyde in OA articular tissues [18]. We recently reported, for the first time, that free HNE induces cartilage degradation in isolated OA chondrocytes and alters the cellular phenotype of OA osteoblasts. These responses are mediated by the modulation of a panoply of signaling pathways, including mitogen-activated protein kinases and NF-κB [18-20]. By binding to proteins, HNE activates MMP-13 and increases the susceptibility of type II collagen to proteolytic cleavage by MMP-13 [18]. In another study, we demonstrated that HNE contributes to inflammatory responses in OA chondrocytes through the transcriptional upregulation of COX-2 via the p38 mitogen-activated protein kinase signaling pathway. We observed that HNE-induced COX-2 declines rapidly after 8 hours of incubation. HNE most probably represents one of the main lipid peroxidation products that can modulate physiological as well as pathological processes, as depicted beautifully in a recent, dedicated review [21].

The objectives of the present project were to investigate whether COX-2 and mPGES-1 downregulation, attributed to HNE depletion, is responsible for the switch from COX-2 and mPGES-1 to 5-LOX and FLAP, and to elucidate the molecular mechanisms underlying their expression in HNE-treated human OA chondrocytes.

## Materials and methods

### Tissue samples

Postsurgery, discarded human OA articular cartilage was obtained from OA patients (mean ± standard deviation age, 67 ± 9 years) who underwent total knee arthroplasty. Informed consent had been obtained from patients with OA for the use of their tissues for research purposes. All patients were evaluated by rheumatologists who followed American College of Rheumatology criteria [22]. The Clinical Research Ethics Committee of the Hôpital du Sacré-Cœur de Montréal, including clinicians, researchers and jurists, approved the study protocol and the use of human articular tissues.

### Chondrocyte culture

OA cartilage (femoral condyles and tibial plateaus) was obtained under aseptic conditions and carefully dissected from the underlying bone in each specimen [23]. OA chondrocytes were extracted by sequential enzymatic digestion with 1 mg/ml pronase (Sigma, Oakville, ON, Canada) for 1 hour at 37°C, and then with 2 mg/ml type IV collagenase (Sigma) for 6 hours in DMEM (Invitrogen, Burlington, ON, Canada) supplemented with 10% heat-inactivated fetal bovine serum (Invitrogen), 100 units/ml penicillin and 100 μg/ml streptomycin (Invitrogen). The cells were seeded at high density in culture flasks at 37°C in a humidified atmosphere of 5% CO_2_/95% air until they were confluent and ready for the experiments. First-passage cells were employed to ensure their phenotype and were seeded at 10^5 ^cells/cm^2 ^in culture tissue plates. The DMEM containing 2% fetal bovine serum and antibiotics was replaced 24 hours before the experiments were performed in this medium with the factors under study for different incubation time periods.

For COX-2 and mPGES-1 studies (up to 24 hours), chondrocytes were treated with a single addition of 10 μM HNE or with repeated treatments by adding 10 μM HNE to the cultures at 0, 2, 4, 6, 8, 10, 12 and 14 hours. For 5-LOX and FLAP studies (up to 72 hours), cells were treated with a single addition of 10 μM HNE in the presence or absence of 50 μM naproxen or 100 μg/ml anti-transforming growth factor-beta 1 (TGFβ1).

### Protein detection by western blotting

Twenty micrograms of total proteins from chondrocyte lysates treated with HNE under the indicated conditions were loaded for discontinuous 4 to 12% SDS-PAGE. Protein transfer, immunodetection and semiquantitative measurements were performed as described previously [18]. The primary antibodies were rabbit anti-COX-2 (Cayman Chemical, Hornby, ON, Canada), anti-mPGES-1 (Cayman Chemical), anti-β-actin (Santa Cruz Biotechnology, Santa Cruz, CA, USA) and anti-Egr-1 (Santa Cruz Biotechnology). After serial washes, primary antibodies were detected by goat anti-rabbit IgG conjugated to horseradish peroxidase (Jackson ImmunoResearch Laboratories, Inc., West Grove, PA, USA). Immunoreactive proteins were detected with SuperSignal blotting substrate (Pierce Biotechnology, Inc., Rockford, IL, USA) and were exposed to clear-blue X-ray film (Pierce).

### RNA extraction and RT-PCR

Total RNA was isolated with TRIzol reagent according to the manufacturer's instructions. RNA was quantitated with the RiboGreen RNA quantitation kit (Molecular Probes, Eugene, OR, USA), dissolved in diethylpyrocarbonate-treated H_2_O, and stored at -80°C until use. One microgram of total RNA was reverse-transcribed with Moloney murine leukemia virus reverse transcriptase (Fermentas, Burlington, ON, Canada), as detailed in the manufacturer's guidelines. One-fiftieth of the reverse transcriptase reaction product was analyzed by traditional PCR or by real-time quantitative PCR. The following sense and antisense specific primers (Bio-Corp, Inc., Montreal, QC, Canada), were tested: human mPGES-1, 5'-GAA GAA GGC CTT TGC CAA C-3' (sense) and 5'-GGA AGA CCA GGA AGT GCA TC-3' (antisense); human 5-LOX, 5'-CTG TTC CTG GGC ATG TAC CC-3' (sense) and 5'-GAC ATC TAT CAG TGG TCG TG-3' (antisense); human FLAP, 5'-AAT GGG AGG AGC TTC CAG AG-3' (sense) and 5'-ACC AAC CCC ATA TTC AGC AG-3' (antisense); and human GAPDH, 5'-CAG AAC ATC ATC CCT GCC TCT-3' (sense) and 5'-GCT TGA CAA AGT GGT CGT TGA G-3' (antisense).

Quantitative PCR analysis was performed in a total volume of 50 μl containing template DNA, 200 nM sense and antisense primers, 25 μl SYBR Green Master Mix (Qiagen, Mississauga, ON, Canada), and 0.5 units uracil-*N*-glycosylase (Epicentre Technologies, Madison, WI, USA). After incubation at 50°C for 2 minutes (uracil-*N*-glycosylase reaction) and at 95°C for 10 minutes (uracil-*N*-glycosylase inactivation and activation of AmpliTaq Gold enzyme), the mixtures were subjected to 40 amplification cycles (15 seconds at 95°C for denaturation and 1 minute for annealing and extension at 60°C). Incorporation of SYBR Green dye into the PCR products was monitored in real time with a Mx3000 real-time PCR system (Stratagene, La Jolla, CA, USA), to determine the threshold cycle (C_t_) at which exponential amplification of PCR products begins. After PCR, dissociation curves were generated with one peak, indicating amplification specificity. A C_t _value was obtained from each amplification curve with the software provided by the manufacturer (Stratagene).

Relative mRNA expression in chondrocytes was quantified according to the ΔΔC_t _method, as detailed in the manufacturer's guidelines (Stratagene). A ΔC_t _value was first calculated by subtracting the C_t _value for the housekeeping gene GAPDH from the C_t _value for each sample. A ΔΔC_t _value was then calculated by subtracting the ΔC_t _value for the controls (unstimulated cells) from the ΔC_t _value for each treatment. Fold changes compared with the controls were then determined by 2^-ΔΔC^_t_. Each PCR generated only the expected specific amplicon, as shown by melting temperature profiles of the final product and gel electrophoresis of the test PCRs. Each PCR was performed in triplicate on two separate occasions for each independent experiment.

### Prostaglandin E_2_, leukotriene B4 and TGF-β1 enzyme immunoassay

After incubation, the culture medium from cultured OA chondrocytes was collected and the PGE_2_, LTB_4 _and TGF-β1 levels were measured with specific commercial kits from Cayman Chemical and R&D Systems (Minneapolis, MN, USA), according to the manufacturer's instructions. Detection sensitivity was 9, 13.7 and 4.6 pg/ml, respectively. All assays were performed in duplicate.

### Plasmids and transient transfection

The human mPGES-1 promoter construct (-538/-28) was a gift from Dr Terry J. Smith (University of California, Los Angeles, CA, USA) [24]. The pEgr-1Mutx3-TK-Luc reporter construct was generously provided by Dr Yuqing E. Chen (Morehouse School of Medicine, Atlanta, GA, USA) [25]. The 5-LOX promoter construct was kindly donated by Dr Dieter Steinhilber (University of Frankfurt, Frankfurt, Germany).

Subconfluent human OA chondrocytes were transiently transfected in 12-well cluster plates with lipofectamine 2000™ reagents (Invitrogen Life Technology, Inc.), according to the manufacturer's protocol. Briefly, transfections were conducted for 6 hours with DNA lipofectamine complexes containing 10 μl lipofectamine reagent, 2 μg DNA plasmid and 0.5 μg pCMV-β-galactosidase (as a transfection efficiency control). In cotransfection experiments the amount of transfected DNA was kept constant using a corresponding empty vector. Using the method of Howcroft and colleagues [26], the transfection efficiency was measured and the average rate of transfected cells was 48%. After washing, the cells were incubated for different incubation periods in the presence or absence of 10 μM HNE (single addition) in fresh DMEM culture medium containing 2% fetal bovine serum in a humidified atmosphere of 95% air/5% CO_2 _at 37°C. Luciferase activity was determined in cell lysate with commercially available kits (Luciferase assay system; Promega Corporation, Madison, WI, USA). The data were normalized to the β-galactosidase level, which was quantified in cell lysate by a specific ELISA (Roche Applied Science, Laval, QC, Canada).

### Statistical analysis

All quantitative results were calculated as the mean ± standard error of the mean. The data were assessed by Student's unpaired *t *test. *P *< 0.05 was considered statistically significant.

## Results

### Single or repeated HNE treatments regulate differently PGE_2 _release and COX-2 protein expression

As described in our previous report [20], COX-2 protein and mRNA expression decreased gradually after 8 hours of chondrocyte incubation with single addition of 10 μM HNE. To examine whether this reduction is attributed to HNE depletion, cells were stimulated with either single or repeated 10 μM HNE treatments for different incubation periods (0 to 24 hours, *n* = 4 independent experiments) as described in Materials and methods.

**Figure 1:**
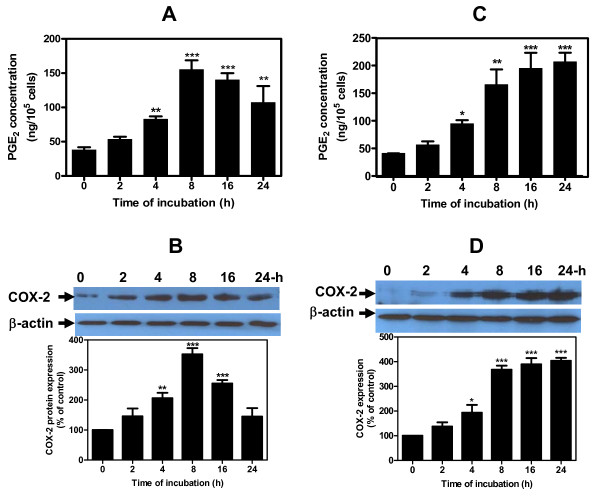
**Single or repeated treatments with hydroxynonenal regulate differently prostaglandin E_2 _release and cyclooxygenase-2 expression**. Human osteoarthritis chondrocytes were treated for different periods of incubation: **(a), (b) **with a single addition of 10 μM 4-hydroxynonenal (HNE), or **(c), (d) **with repeated treatments by adding 10 μM HNE to the cultures at 2-hour intervals up to 14 hours. (a), (c) Prostaglandin E_2 _(PGE_2_) concentrations were measured by enzyme immunoassay commercial kit. (b), (d) Cyclooxygenase-2 (COX-2) protein expression was analyzed by western blotting. Data are means ± standard errors of the mean, n = 4, expressed as percentages of untreated cells. Student's unpaired *t *test: *P < 0.05, **P < 0.01, ***P < 0.001.

Single treatment of chondrocytes with 10 μM HNE showed an increase in COX-2 protein expression and PGE_2 _release within 2 hours and plateaued at 8 hours of incubation, before declining at 16 hours and 24 hours (Figure 1a, b). PGE_2 _and COX-2 reached maximum levels of 157.5 ± 33 ng/10^5 ^cells and 340% of control values, respectively (*P *< 0.001). In experiments where the cells were stimulated with repetitive addition of 10 μM HNE, however, PGE_2 _release and COX-2 protein expression were enhanced after 4 hours of incubation, with maximum values obtained at 24 hours (Figure 1c, d). Maximum PGE_2 _and COX-2 values were 210 ± 29 ng/10^5 ^cells and 400% of control values, respectively (*P *< 0.001). Single or repeated treatments with 10 μM HNE did not induce either toxic or apoptotic phenomena in chondrocytes (data not included).

These data suggest for the first time that repeated treatments at 2-hour intervals with 10 μM HNE are essential to maintain the protein expression of COX-2 up to 24 hours.

### Induction of mPGES-1 protein and mRNA expression by single or repeated HNE treatments

To gain insights into the role of HNE in the mechanism underlying the production of PGE_2 _we evaluated its effect on the protein and mRNA expression of mPGES-1, an inducible form of PGES involved in the terminal step of PGE_2 _synthesis. Human OA chondrocytes were treated once or several times for different incubation periods as described in Materials and methods (*n* = 4 independent experiments).

**Figure 2:**
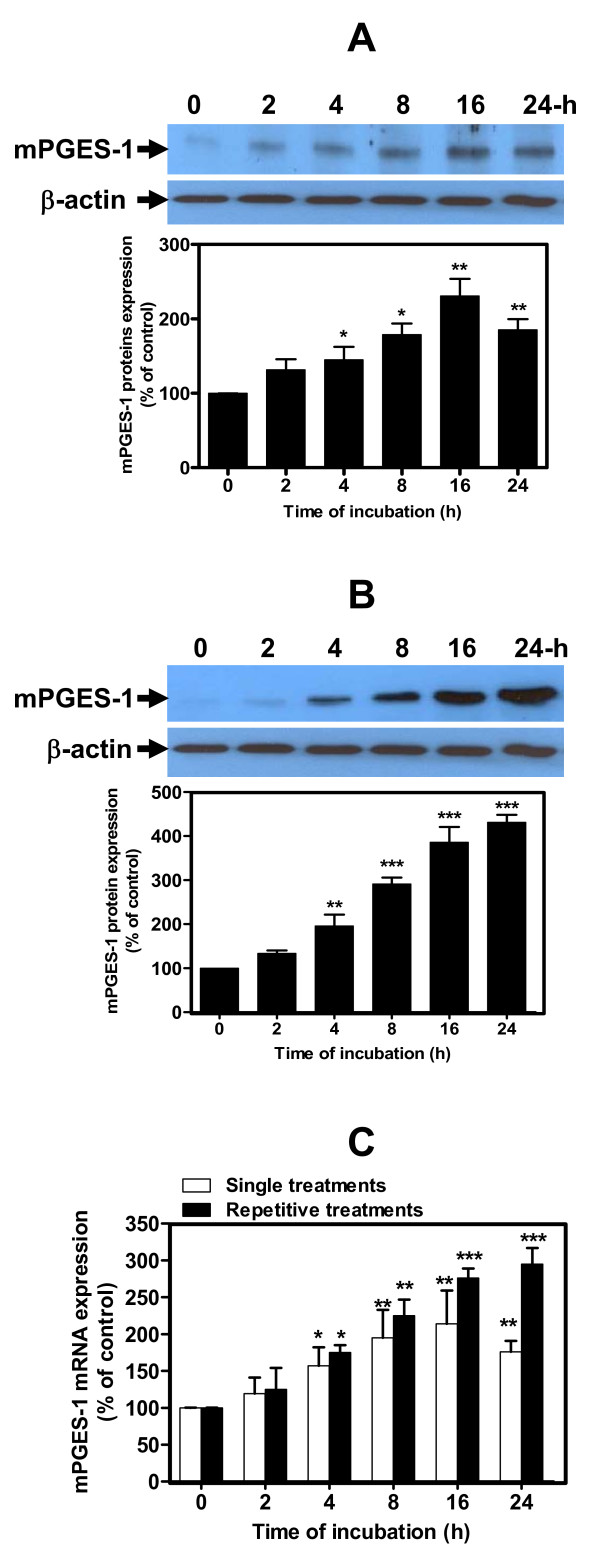
**Hydroxynonenal-induced microsomal prostaglandin E_2 _synthase-1 protein and mRNA expression in human osteoarthritis chondrocytes**. Cells were stimulated **(a), (c) **with 10 μM 4-hydroxynonenal (HNE) once or **(b), (c) **repeatedly with 10 μM HNE every 2 hours for different incubation periods, as indicated above. Microsomal prostaglandin E_2 _synthase-1 (mPGES-1) **(a), (b) **protein and **(c) **mRNA were measured respectively by western blotting and real-time RT-PCR. Data are means ± standard errors of the mean, n = 4, expressed as percentages of untreated cells. Student's unpaired *t *test: *P < 0.05, **P < 0.01, ***P < 0.001.

Treatment of chondrocytes with single addition of 10 μM HNE to cultures significantly increased mPGES-1 protein and mRNA levels, which plateaued at 16 hours but then declined at 24 hours (Figure 2a, c). mPGES-1 protein and mRNA reached maximum levels of 230 and 220% of control values, respectively (*P *< 0.01). Repeated treatments at 2-hour intervals with 10 μM HNE, however, enhanced mPGES-1 protein and mRNA levels and plateaued at 24 hours (Figure 2b, c). The maximum levels of mPGES-1 protein and mRNA were 400 and 300% of the controls, respectively (*P *< 0.001).

Similar to COX-2, our data indicate that the maintained mPGES-1 protein and mRNA expression might be linked to repetitive treatments with HNE.

### HNE-induced mPGES-1 and 3xEgr-1 binding site promoter activities

In our previous report, we reported that HNE induced COX-2 transcription through ATF-2/CREB-1 transactivation [20]. In the present study, we further examined whether HNE induced mPGES-1 at a transcriptional level through Egr-1 transactivation.

**Figure 3:**
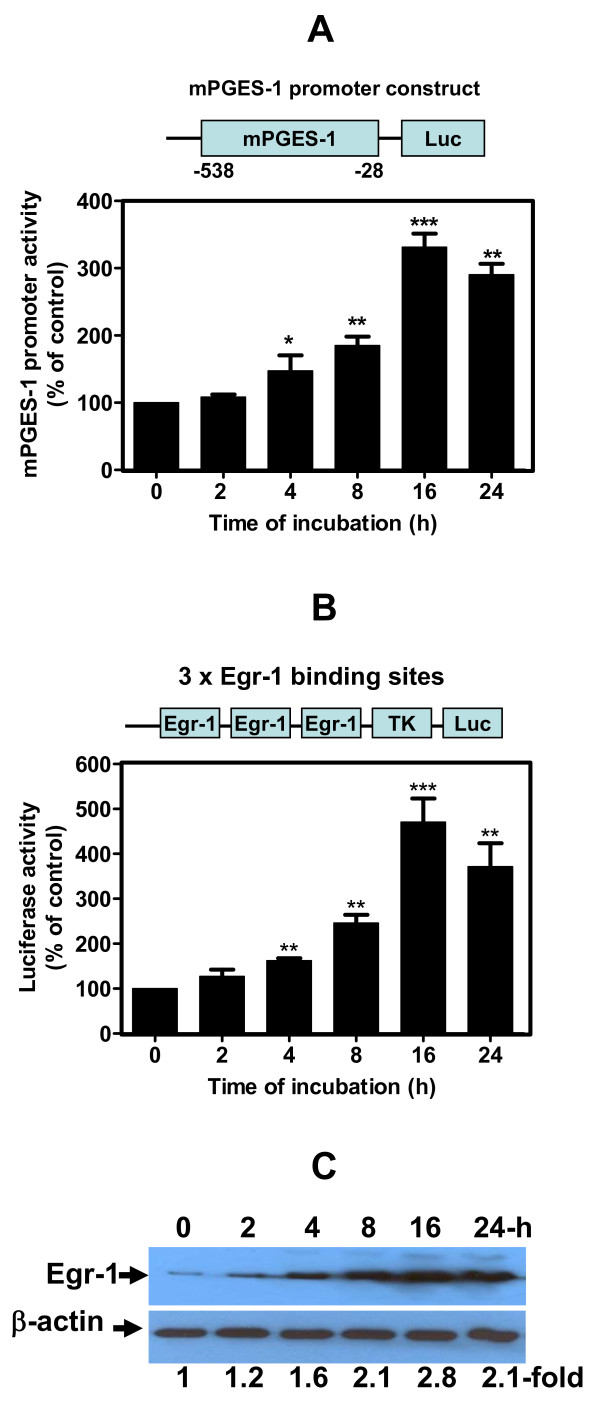
**Hydroxynonenal-activated microsomal prostaglandin E_2 _synthase-1 promoter**. Chondrocytes were transfected with **(a) **microsomal prostaglandin E_2 _synthase-1 (mPGES-1)-Luc or **(b) **p3xEgr-1-Luc containing three early growth response factor 1 (Egr-1) binding sites. The total amount of transfected DNA was kept constant using a corresponding empty vector. The next day, they were incubated with single addition of 10 μM 4-hydroxynonenal (HNE) for increasing incubation time periods. Luciferase activity was measured in cell extracts and normalized to β-galactosidase activity. **(c) **Osteoarthritis chondrocytes were treated with 10 μM HNE at increasing time of incubation and then Egr-1 protein expression was evaluated in the cellular extract by western blot. Data are means ± standard errors of the mean, n = 6, expressed as percentages of untreated cells. Student's unpaired *t *test: *P < 0.05, **P < 0.01, ***P < 0.001.

Firstly, to determine whether changes in mPGES-1 mRNA levels can be ascribed to the regulation in promoter activity, chondrocytes were transiently transfected with human mPGES-1 promoter-luciferase reporter genes (*n* = 6 independent experiments) and then treated with single addition of 10 μM HNE to all wells except the controls. As shown in Figure 3a, HNE induced mPGES-1 promoter activity in a time-dependent increment. Maximum promoter activity was 331% at 16 hours compared with the control (*P *< 0.001).

Secondly, we analyzed the effect of HNE on the transcriptional activation of a synthetic luciferase reporter construct containing three tandem repeats of the putative Egr-1 binding sequence, pEgr-1x3-TK-Luc. The transcription factor Egr-1 is believed to mediate the induction of a host of target genes such as mPGES-1, as suggested by Cheng and colleagues [8]. As seen in Figure 3b, HNE increased the luciferase activity of the above construct, and this activation was time dependent. Maximum promoter activity reached 450% at 16 hours compared with the controls (*P *< 0.001). Western blot analysis revealed that single treatment with 10 μM HNE induced Egr-1 protein expression by 280% compared with untreated cells after 16 hours of incubation (Figure 3c).

Collectively, these data suggest that Egr-1 may be one of the transcription factors involved in HNE-induced mPGES-1 promoter activity.

### HNE-induced LTB4 release through 5-LOX and FLAP upregulation

**Figure 4:**
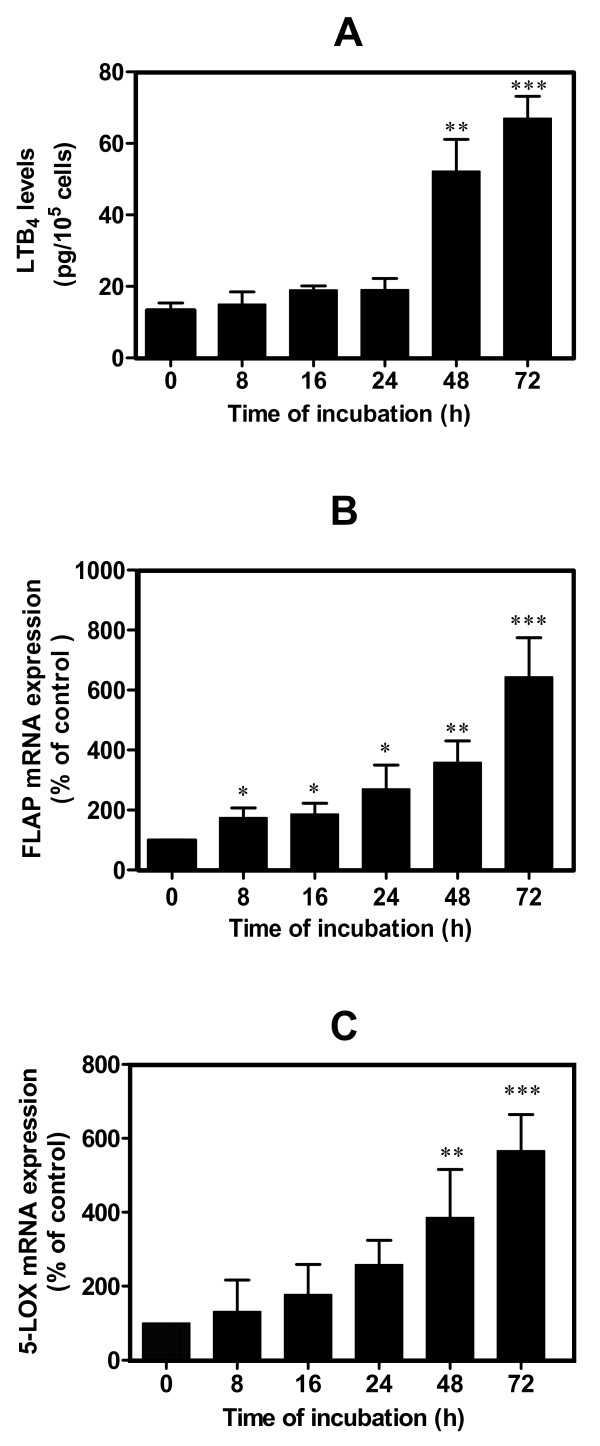
**Hydroxynonenal-induced leukotriene B_4 _biosynthesis and 5-lipoxygenase and 5-lipoxygenase-activating protein expression in human osteoarthritis chondrocytes**. Cells were treated with single addition of 10 μM 4-hydroxynonenal for different incubation time periods, as indicated above. **(a) **Leukotriene B_4 _(LTB_4_) production was evaluated by enzyme immunoassay commercial kit. **(b) **5-lipoxygenase-activating protein (FLAP) and **(c) **5-lipoxygenase (5-LOX) mRNA expression was measured by real-time RT-PCR. Data are means ± standard errors of the mean, n = 5, expressed as percentages of untreated cells. Student's unpaired *t *test: *P < 0.05, **P < 0.01, ***P < 0.001.

In the next series of experiments, we further investigated whether the decrease of COX-2 expression in treated chondrocytes with single addition of 10 μM HNE to cultures lead to a switch to 5-LOX and FLAP mRNA expression, and consequently to LTB_4 _production. LTB_4 _is a potent chemoattractant and stimulator of inflammation [27] and probably contributes, along with other factors, to the upregulation of the synthesis of other catabolic factors involved in the pathophysiology of OA [28]. Human OA chondrocytes (*n* = 5 independent experiments) were treated for different incubation periods with single addition of 10 μM HNE to all wells except the controls. HNE-induced changes in the LTB_4 _level and in 5-LOX and FLAP mRNA expression and were evaluated respectively in picograms per milliliter or as a percentage of control. LTB_4 _release (Figure 4a) was induced only after a long incubation period. The maximum level rose significantly after 72 hours of treatment (66.6 ± 3.7 pg/10^5 ^cells, *P *< 0.001).

5-LOX and FLAP are essential proteins responsible for LTB_4 _synthesis. To determine whether augmented LTB_4 _production induced by HNE is related to 5-LOX and FLAP synthesis, we further examined the effect of HNE on their mRNA expression. As shown in Figure 4b, c, our data disclosed that both FLAP and 5-LOX mRNA expression rose significantly but differentially after incubation in human OA chondrocytes. FLAP mRNA expression occurred earlier, after 8 hours of incubation, compared with 5-LOX (48 hours). Maximum FLAP and 5-LOX levels were 630% and 560% of control values, respectively (*P *< 0.001).

Taken together, these results indicate that single treatment with HNE in OA chondrocytes induced the shunt from the production of PGE_2 _to LTB_4 _through COX-2 downregulation and 5-LOX and FLAP upregulation.

### HNE-induced 5-LOX promoter activity

**Figure 5:**
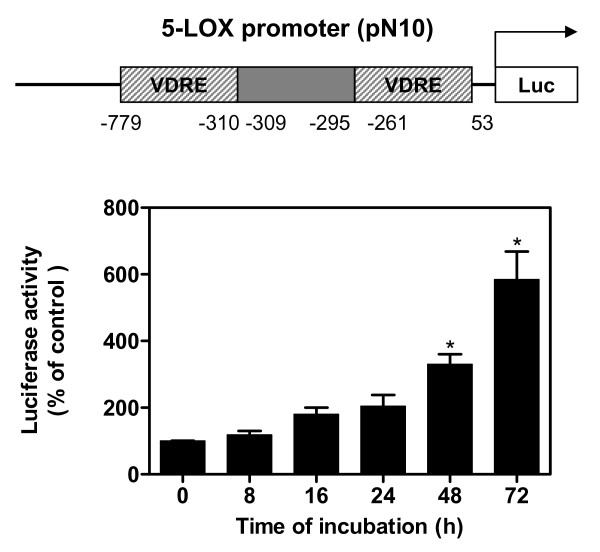
**Hydroxynonenal-activated 5-lipoxygenase promoter in human osteoarthritis chondrocytes**. Cells were transfected with human 5-lipoxygenase (5-LOX) promoter. The next day, they were incubated with single addition of 10 μM 4-hydroxynonenal for increasing incubation time periods. Luciferase activity was measured in cell extracts and normalized to β-galactosidase activity. Data are means ± standard errors of the mean, n = 4, expressed as percentages of untreated cells. Student's unpaired *t *test: *P < 0.05. VDRE: vitamin D responsive element.

To more accurately identify whether HNE induced 5-LOX at the transcription level, the 5-LOX promoter construct with luciferase gene reporter was transiently transfected into human OA chondrocytes, followed by stimulation with single addition of 10 μM HNE to all wells excepted control for different time periods (*n* = 4 independent experiments). Compared with unstimulated cells, HNE induced 5-LOX gene promoter activity in transfected chondrocytes. 5-LOX promoter activity increased significantly by 310% and 580% after 48 and 72 hours of incubation, respectively (*P *< 0.05) (Figure 5).

These data indicate that HNE regulates 5-LOX mRNA expression at the transcriptional level.

### HNE-induced 5-LOX and FLAP expression is PGE_2 _and TGF-β1 dependent

**Figure 6:**
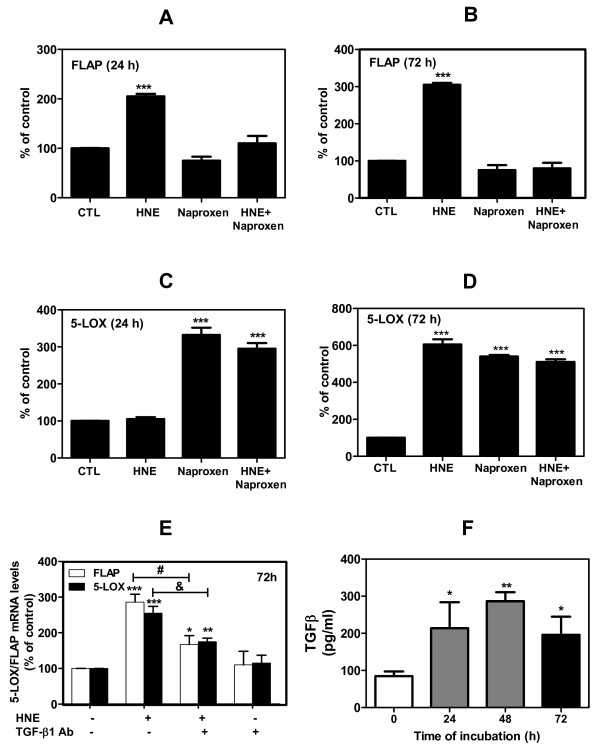
**Hydroxynonenal-induced 5-lipoxygenase and 5-lipoxygenase-activating protein expression is prostaglandin E_2_-dependent and TGF-β-dependent in human osteoarthritis chondrocytes**. Cells were treated with 50 μM naproxen, 10 μM 4-hydroxynonenal (HNE) and 50 μM naproxen + 10 μM HNE for 24 or 72 hours. **(a), (b) **5-Lipoxygenase-activating protein (FLAP) and **(c), (d) **5-lipoxygenase (5-LOX) mRNA expression at 24 and 72 hours was evaluated by real-time RT-PCR. **(e) **Cells were treated with single addition of 10 μM HNE for 72 hours in the presence or absence of anti-TGF-β1 antibody, and 5-LOX and FLAP mRNA levels were then assessed by real-time RT-PCR (^#^*P *< 0.01 and ^&^*P *< 0.05, respectively). **(f) **Cells were treated with single addition of 10 μM HNE for 24, 48, and 72 hours, and the transforming growth factor-beta 1 (TGF-β1) level was measured with ELISA commercial kits and expressed as picograms per milliliter. Data are means ± standard errors of the mean, n = 4, expressed as percentages of untreated cells. Student's unpaired *t *test: ***P < 0.001. Ab: antibody; CTL: control.

Further experiments were aimed at elucidating the role of PGE_2 _and TGF-β1 production in HNE-induced 5-LOX and FLAP expression in OA chondrocytes. Firstly, cells were treated with 50 μM naproxen (a nonselective COX inhibitor) for 1 hour followed by another treatment for 24 or 72 hours with single addition of 10 μM HNE to all wells except the controls (*n* = 4 independent experiments). HNE-induced changes in 5-LOX and FLAP mRNA expression were evaluated as a percentage of control. Our real-time RT-PCR results demonstrated that HNE induced FLAP expression after 24 hours of treatment (210% of control, *P *< 0.001) and the level was even higher after 72 hours (315% of control, *P *< 0.001) (Figure 6a, b). FLAP activation by HNE was abolished by naproxen, which prevents PGE_2 _production. In contrast, HNE induced 5-LOX expression by 600% above control values (*P *< 0.001) (Figure 6d) only after 72 hours of incubation. Incubation of cells with naproxen, however, caused a consistent increase of 5-LOX mRNA level by 330% and 570% of control values (*P *< 0.001) after 24 and 72 hours of incubation, respectively (Figure 6c, d). The addition of naproxen to HNE had no additive effect on the 5-LOX mRNA level. All these data suggest that PGE_2 _may play a critical role in 5-LOX and FLAP regulation by HNE.

Secondly, to determine whether TGF-β1 production could be involved in HNE-induced 5-LOX and FLAP expression, chondrocytes were treated or not with a single addition of 10 μM HNE for 72 hours in the presence or absence of 100 μg/ml TGF-β1 antibody. HNE-induced changes in 5-LOX and FLAP mRNA expression were evaluated as a percentage of control (untreated cells). As illustrated in Figure 6e, the addition of anti-TGF-β1 antibody to cultured cells attenuated HNE-induced 5-LOX and FLAP by 40% (both *P *< 0.05). In another experiment, the determination of TGF-β1 by ELISA in the culture media from HNE-treated chondrocytes showed a significant increase in TGF-β1 levels in comparison with untreated cells (control), and reached 210, 288 and 201 pg/ml after 24, 48, and 72 hours of incubation, respectively (Figure 6f) (*n* = 5 independent experiments, *P *< 0.05, *P *< 0.01 and *P *< 0.05).

Collectively, our results indicate that HNE-induced 5-LOX and FLAP requires PGE_2 _and TGF-β1 production.

## Discussion

HNE, a product of lipid peroxidation, has been identified as a modulator of signal transduction, gene transcription and protein modification [21]. Our previous study demonstrated that the level of HNE is significantly higher in synovial fluids and in chondrocytes of OA patients compared with normal subjects. HNE induces cartilage degradation by transcriptional and post-transcriptional changes of type II collagen and MMP-13 in OA [18]. Recently, HNE was found to be an inflammatory factor that stimulates the production of proinflammatory mediators, including PGE_2_, but its role in the pathology of joint and inflammatory diseases remains unclear. In the present study, we investigated the effects of HNE on COX-2/mPGES-1 and 5-LOX pathways regulation in human OA chondrocytes. COX-2/mPGES-1 and 5-LOX are primary proteins in the synthesis of PGE_2 _and LTB_4_, respectively.

In the present study, we observed that single treatment of OA chondrocytes with 10 μM HNE induced PGE_2 _release as well as COX-2 and mPGES-1 expression, which plateaued at 8 hours of incubation before declining at 16 and 24 hours, respectively. These data are in concordance with our previously reported COX-2 findings [20] and are supported by other investigations indicating that HNE is a potent inducer of COX-2 and PGE_2 _release in various cell lines, such as rat liver epithelial RL34 cells and the RAW264.7 macrophage cell line [29-31]. Interestingly, when HNE was maintained for 14 hours in cultured chondrocytes, through repeated treatments at intervals of 2 hours, the aldehyde induced both COX-2 and mPGES-1 expression up to 24 hours and consequently abolished their decrease. It has been shown that the maintaining of HNE levels at 1 μM through repetitive treatments regulated cell proliferation and differentiation [32]. Our observations support the view that decreased COX-2 and mPGES-1 expression is attributed to HNE depletion via its metabolism in chondrocytes. Mammalian cells have developed multiple enzymatic pathways for HNE detoxification. The best characterized of these enzymes include glutathione S-transferases, aldehyde dehydrogenase, and alcohol dehydrogenase [33]. The half-life of HNE in cells was estimated to be ~1 hour [34]. On the contrary, we do not exclude the possibility that the increase of COX-2 and mPGES-1 expression up to 24 hours may coincide with the inflammatory resolution. As demonstrated by Gilroy and colleagues [35], COX-2 plays a role as a proinflammatory factor in the early phase of inflammation. During the late phase, however, this enzyme regulates resolution of acute inflammation by generating an alternate set of prostaglandins, such as the cyclopentenone family.

Since mPGES-1 regulation by HNE has not yet been reported, we extended our investigation to determine whether changes in mRNA levels may be ascribed to alterations in promoter activity by transiently transfecting chondrocytes with human mPGES-1 promoter-luciferase reporter genes. The treatment of transfected cells with single addition of 10 μM HNE to cultures led to a time-dependent increment of mPGES-1 promoter activity. These data are consistent with the regulation of mPGES-1 expression by HNE at the transcription level. Emerging evidence has disclosed that the transcriptional induction of mPGES-1 is primarily controlled by Egr-1 through two Egr-1 binding motifs identified in the proximal promoter region of mPGES-1 [36,37]. Numerous cytokines and growth factors are known to upregulate mPGES-1 production via Egr-1 activation [38]. We hypothesized that the induction of Egr-1 activity by HNE could be the mechanism by which HNE exerts its stimulatory effect on mPGES-1 transcription. In transfected cells, HNE evoked the activation of a synthetic luciferase reporter construct containing three tandem repeats of Egr-1 motif. Our western blot data demonstrated that 10 μM HNE heightened Egr-1 protein expression in a time-dependent manner. Collectively, these data suggest the involvement of this transcription factor in HNE-induced transcriptional activity of the mPGES-1 promoter in OA chondrocytes. Further experiments are needed, however, to confirm the direct implication of this transcription factor in HNE-induced mPGES-1.

Thereafter, we explored 5-LOX and FLAP regulation in human OA chondrocytes by HNE, examined the relative involvement of these two proteins in LTB_4 _production, and studied the factors that might be responsible for enhanced LTB_4 _production in these cells incubated for long periods. Our data indicated that 5-LOX and FLAP upregulation by HNE occurred, at least in part, at the transcription level, as determined by real-time quantitative RT-PCR and transient transfection experiments. HNE-induced 5-LOX and FLAP gene activation is differentially time dependent. FLAP expression is activated earlier than 5-LOX, which occurs only after 48 hours of HNE stimulation. These results are consistent with a previous report of Martel-Pelletier and colleagues showing that 5-LOX expression is activated after that of FLAP, leading to the late increase of LTB_4 _production [28]. The FLAP mRNA level was significantly enhanced after a short period (20 hours) of treatment with TGF-β1 or 1,25-dihydroxyvitamin D_3 _alone or combined, whereas the 5-LOX mRNA level rose only after 72 hours [28]. These authors postulated that the reason for the late increment of 5-LOX mRNA may be that TGF-β1 induces only 5-LOX mRNA accumulation and not true upregulation of gene expression *per se*. Heightened LTB_4 _production upon HNE stimulation occurred 48 hours after the beginning of stimulation, coinciding with the time at which the 5-LOX mRNA level was elevated, and FLAP gene expression remained high. These data confirm that LTB_4 _production is dependent on the regulation of both 5-LOX and FLAP. Other studies, however, have demonstrated that the increase in LTB_4 _production was mainly related to upregulation of the FLAP gene [39] or to a combination of an increment in the activity and/or expression of 5-LOX [40,41].

Several investigations have shown that 5-LOX and FLAP expression is regulated by PGE_2 _[28,42]. Our results put this observation in evidence. PGE_2 _inhibition regulates 5-LOX and FLAP expression differentially. Using the nonspecific inhibitor of COX, naproxen, we abolished HNE-induced FLAP expression in OA chondrocytes after short or long periods of incubation. In contrast, naproxen induced 5-LOX expression after short or long stimulation time periods. The combination of naproxen with HNE, however, had no additive effect on 5-LOX mRNA expression. Significant advances have been made in understanding the role of PGE_2 _in the metabolism of articular tissues. PGE_2 _has been found not only to be involved in inflammatory responses but may also regulate the effects of other inflammatory mediators. More pharmaceutical companies therefore focus on mPGES-1 to treat arthritis because of its inducible form and functional linkage with COX-2 [38].

Current therapies to treat OA are limited to reducing the pain in affected joints by with either nonselective nonsteroidal anti-inflammatory drugs or selective COX-2 agents. The production of both PGE_2 _and LTB_4 _requires AA; therefore, one of the pathways is used favorably, depending on the condition. Long-term COX inhibition causes the switch from COX to the 5-LOX pathway. This is observed in OA patients treated with COX-2 inhibitors [43]. In our study, we also demonstrated that lower PGE_2 _production favors the 5-LOX pathway and LTB_4 _synthesis in HNE-treated OA chondrocytes. Leukotrienes have greater potential in the inflammatory process, however, and may be more harmful than PGE_2 _in various tissues due to their chemotactic properties [44]. For this reason, drugs that can inhibit both COX and lipoxygenase have attracted pharmaceutical attention in the treatment of inflammatory diseases. Tepoxalin and licofelone (ML3000) have been identified to exert this function [45,46]. Today, licofelone is in phase III clinical development. Licofelone not only lowers PGE_2 _and LTB_4 _production, but can also modify abnormal bone remodeling in OA; thus, it was thought to be useful in treating OA [46].

In addition to the involvement of PGE_2 _in HNE-induced 5-LOX and FLAP, we found data supporting the role of TGF-β1 in this process. Our observations showed that HNE evoked TGF-β1 synthesis and that TGF-β1 neutralization by anti-TGF-β1 antibody attenuated HNE-induced 5-LOX and FLAP after 72 hours of incubation. These results suggest that 5-LOX and FLAP regulation by HNE is partially mediated via TGF-β1 production. Leonarduzzi and colleagues were the first to demonstrate that HNE stimulated TGF-β1 production [47]. Furthermore, a number of reports support the role of TGF-β1 in 5-LOX and FLAP expression through smad3/4 activation [13,28,40].

## Conclusions

**Figure 7:**
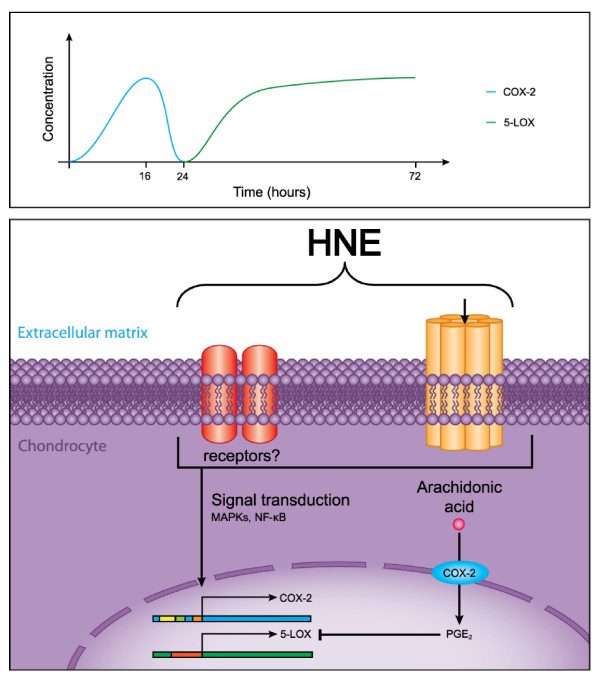
**Overview of the regulation of cyclooxygenase-2 and 5-lipoxygenase expression by 4-hydroxynonenal**. In short-term of incubation, HNE induces a panoply of signaling pathways-mediating PGE_2 _production through COX-2 upregulation. PGE_2 _negatively regulates 5-LOX and LTB_4 _productions. In long-term of incubation, the increase of 5-LOX and LTB_4 _levels could be due to a decrease in COX-2 and PGE_2 _levels caused by gradual HNE depletion. COX-2: cyclooxygenase-2; HNE: 4-hydroxynonenal; 5-LOX: 5-lipoxygenase; MAPK: mitogen-activated protein kinase; PGE_2_: prostaglandin E_2_.

Our data provide new evidence for the inflammatory role of HNE, an oxidative stress-related product, in the physiopathology of OA. As illustrated in Figure 7, our findings suggest that COX-2 and mPGES-1 downregulation is attributed to HNE depletion and may be responsible for the switch from COX-2 to 5-LOX and FLAP pathways. Both PGE_2 _and TGF-β1 are involved in the regulation of 5-LOX and FLAP by HNE in OA chondrocytes. In this context, a drug that inhibits HNE production may be beneficial to attenuate inflammation in the treatment of OA and inflammatory diseases.

## Abbreviations

AA: arachidonic acid; COX: cyclooxygenase; C_t_: threshold cycle; DMEM: Dulbecco's modified Eagle's medium; Egr-1: early growth response factor 1; ELISA: enzyme-linked immunosorbent assay; FLAP: 5-lipoxygenase-activating protein; HNE: 4-hydroxynonenal; IL: interleukin; 5-LOX: 5-lipoxygenase; LTB_4_: leukotriene B_4_; MMP: metalloproteinase; mPGES-1: microsomal prostaglandin E_2 _synthase-1; NF: nuclear factor; OA: osteoarthritis; PCR: polymerase chain reaction; PGE_2_: prostaglandin E_2_; PGES: prostaglandin E_2 _synthase; RT: reverse transcription; TGF-β1: transforming growth factor-beta 1; TNF: tumor necrosis factor.

## Competing interests

The authors declare that they have no competing interests.

## Authors' contributions

S-HC performed the experimental study, contributed to preparation of the manuscript and undertook the statistical analysis. HF evaluated and interpreted the data and assisted with preparation of the manuscript. QS assisted in the experiments and in the isolation of chondrocytes from human cartilage. MB designed the study, supervised the project, evaluated and interpreted the data, and prepared the manuscript. All authors read and approved the final manuscript.
